# Improving screening, treatment, and intervention for unhealthy alcohol use in primary care through clinic, practice-based research network, and health plan partnerships: Protocol of the ANTECEDENT study

**DOI:** 10.1371/journal.pone.0269635

**Published:** 2022-06-28

**Authors:** Amrita N. Singh, Victoria Sanchez, Erin S. Kenzie, Eliana Sullivan, James L. McCormack, Jean Hiebert Larson, Alissa Robbins, Tiffany Weekley, Brigit A. Hatch, Caitlin Dickinson, Nancy C. Elder, John P. Muench, Melinda M. Davis

**Affiliations:** 1 Oregon Rural Practice-Based Research Network, Oregon Health & Science University, Portland, Oregon, United States of America; 2 Oregon Health Authority Transformation Center, Portland, Oregon, United States of America; 3 Department of Family Medicine, Oregon Health & Science University, Portland, Oregon, United States of America; 4 Community Health, OHSU-PSU School of Public Health, Portland, Oregon, United States of America; Public Library of Science, UNITED KINGDOM

## Abstract

**Background:**

Unhealthy alcohol use (UAU) is a leading cause of morbidity and mortality in the United States, contributing to 95,000 deaths annually. When offered in primary care, screening, brief intervention, referral to treatment (SBIRT), and medication-assisted treatment for alcohol use disorder (MAUD) can effectively address UAU. However, these interventions are not yet routine in primary care clinics. Therefore, our study evaluates tailored implementation support to increase SBIRT and MAUD in primary care.

**Methods:**

ANTECEDENT is a pragmatic implementation study designed to support 150 primary care clinics in Oregon adopting and optimizing SBIRT and MAUD workflows to address UAU. The study is a partnership between the Oregon Health Authority Transformation Center—state leaders in Medicaid health system transformation—SBIRT Oregon and the Oregon Rural Practice-based Research Network. We recruited clinics providing primary care in Oregon and prioritized reaching clinics that were small to medium in size (<10 providers). All participating clinics receive foundational support (i.e., a baseline assessment, exit assessment, and access to the online SBIRT Oregon materials) and may opt to receive tailored implementation support delivered by a practice facilitator over 12 months. Tailored implementation support is designed to address identified needs and may include health information technology support, peer-to-peer learning, workflow mapping, or expert consultation via academic detailing. The study aims are to 1) engage, recruit, and conduct needs assessments with 150 primary care clinics and their regional Medicaid health plans called Coordinated Care Organizations within the state of Oregon, 2) implement and evaluate the impact of foundational and supplemental implementation support on clinic change in SBIRT and MAUD, and 3) describe how practice facilitators tailor implementation support based on context and personal expertise. Our convergent parallel mixed-methods analysis uses RE-AIM (reach, effectiveness, adoption, implementation, maintenance). It is informed by a hybrid of the i-PARIHS (integrated Promoting Action on Research Implementation in Health Services) and the Dynamic Sustainability Framework.

**Discussion:**

This study will explore how primary care clinics implement SBIRT and MAUD in routine practice and how practice facilitators vary implementation support across diverse clinic settings. Findings will inform how to effectively align implementation support to context, advance our understanding of practice facilitator skill development over time, and ultimately improve detection and treatment of UAU across diverse primary care clinics.

## Introduction

The societal and economic costs of unhealthy alcohol use (UAU) are well known [1] and continue to worsen. Between 1999 and 2017, annual alcohol-related deaths among people over the age of 15 doubled in the U.S. [2]. Unhealthy alcohol use and its sequelae worsened even further during the COVID-19 pandemic, with a recent study finding that alcohol-related deaths increased 25% in 2020 compared to 2019 [3, 4].

Much of this morbidity and mortality is preventable. Screening for UAU and intervening with behavioral counseling [5] or medication [6] in the primary care setting are effective measures to reduce alcohol-related problems at individual and population levels. Screening, brief intervention, and referral to treatment (SBIRT) is a clinical process that systematizes the use of validated screening tools and provides brief advice or motivational interviewing to help patients see gaps between their health goals and current behavior [7]. If the screening and further evaluation reveal a more severe use disorder, clinicians can refer patients to specialty treatment or provide medication-assisted treatment within the primary care setting.

Despite clear evidence demonstrating the effectiveness of SBIRT over the past 50 years, SBIRT continues to be inadequately performed in primary care settings [8]. Evaluation of 2017 U.S Behavioral Risk Factor Surveillance System data showed that only 37.8% of adults remembered being asked about binge drinking during their last check-up; of those who reported binge drinking behaviors, only 41.7% were advised about the harms of this behavior, and only 20.1% were advised to reduce or quit [9]. Similarly, medication-assisted treatment for alcohol use disorder (MAUD) is prescribed to fewer than 9% of patients likely to benefit from it [6]. Adaptation of SBIRT to meet clinic and patient needs has the potential to effectively reduce UAU in a clinic population while minimizing its impact on other clinical workflows [10]. However, questions remain regarding best practices when implementing evidence-based interventions for screening, brief intervention, and MAUD in primary care [11].

Therefore, ANTECEDENT (p**A**rt**N**erships **T**o **E**nhance al**C**ohol scr**E**ening, treatment, an**D** int**E**rve**NT**ion) is designed to help primary care clinics implement and refine workflows to support SBIRT and MAUD during routine care. Study activities leverage the infrastructure of an established practice-based research network, the state public health authority, and the previously constructed “SBIRT Oregon” online toolkit [12]. Implementation support is delivered using practice facilitation as a central and unifying strategy to enhance the adoption, implementation, and sustainability of SBIRT and MAUD while aligning implementation strategies to context [13, 14]. Practice facilitation is an implementation strategy in which trained facilitators support capacity development by working with clinic leaders and staff to implement evidence-based practices [15–17]. Practice facilitators use a variety of strategies to assist with goal setting and attainment, connect teams to system-level resources for change, and improve efficiency and team dynamics around improvement processes [16]. The overarching goal of ANTECEDENT is to increase the standard delivery of screening and intervention for UAU in primary care while gaining insight into how and why practice facilitators tailor implementation support.

## Materials and methods

This pragmatic implementation study bridges improvement science and implementation science, allowing for local change and the production of generalizable knowledge [18]. Our convergent parallel mixed-methods design [19, 20] utilizes quantitative, qualitative, and systems science approaches to address the research aims. ANTECEDENT is one of six awards funded in September 2019 by the Agency for Health Care Research and Quality (AHRQ) as part of the EvidenceNOW Managing Unhealthy Alcohol Use Initiative [21]. Study activities were designed to improve SBIRT and MAUD in 150 primary care clinics via 12 months of implementation support coordinated by a trained practice facilitator. The RE-AIM (reach, effectiveness, adoption, implementation, maintenance) model provides a structure for the mixed-methods evaluation. Study activities are approved by the Oregon Health & Sciences University Institutional Review Board (IRB) through an expedited review (STUDY00020592). The protocol is described as of February 2020, before initiating study activities with participating clinics or the onset of COVID-19 research and practice restrictions.

### Study leadership and setting

ANTECEDENT is a partnership between the Oregon Rural Practice-based Research Network (ORPRN), SBIRT Oregon, and the Oregon Health Authority (OHA) Transformation Center. ORPRN is a practice-based research network (PBRN) established in 2002 with a mission to “improve health outcomes, and equity for all Oregonians through community partnered dialogue, research, coaching and education” [18, 22, 23]. PBRNs play an important role in the United States and internationally in bridging research discoveries and routine practice [24, 25]. ORPRN leverages statewide relationships with clinic and community partners, regionally distributed practice facilitators, and a mixed-methods evaluation team to support study activities. SBIRT Oregon (www.sbirtoregon.org) is an established online toolkit designed to support training and implementation of the SBIRT process in clinical practice. The website includes multiple tools and resources (e.g., demonstration videos, clinical workflow guides) to increase SBIRT and MAUD and empower clinicians in primary care to act on positive screening for unhealthy substance use [12]. The OHA Transformation Center oversees many aspects of the implementation and evaluation of Oregon’s regional Medicaid Coordinated Care Organizations (CCOs) and provides technical assistance to support transformation efforts [26]. OHA introduced SBIRT for unhealthy alcohol and drug use as an annual quality metric for Oregon CCOs in 2013; the SBIRT metric has been reported in multiple years since (S1 Appendix) [27]. ANTECEDENT study activities were designed to align with the CCO SBIRT quality incentive metric in collaboration with OHA and the 15 CCOs operating in Oregon at grant submission [28].

### Specific aims and study timeline

The aims of ANTECEDENT are to:

1. Engage, recruit, and conduct needs assessments with 150 primary care clinics and their regional CCOs within the state of Oregon.
2. Implement and evaluate the impact of foundational and supplemental implementation support on SBIRT, MAUD, and quality improvement (QI) capacity in participating primary care clinics.
3. Describe how practice facilitators tailor implementation support based on context, intervention, and personal expertise using mixed-methods and systems science.

Table 1 summarizes the specific aims, research questions, and data sources for ANTECEDENT. Study activities were designed to support engagement and analysis to address the research aims within three years (S1 Table). During grant preparation, we strategically chose not to randomize clinics to different implementation waves or treatment arms and instead allowed clinics to select their preferred quarter to initiate study activities. We anticipate that this approach would optimize a clinic’s ability to engage in support and study evaluation activities. As described by Curran and colleagues, implementation study designs are appropriate after an adequate body of evidence has accumulated that established the efficacy and effectiveness of the intervention [29], as is the case for SBIRT and MAUD [5, 30, 31]. While randomized control trials are often considered the gold standard, they inadequately address “wicked” problems [32] that require attention to individual-, clinic-, and system-level factors and their emergent properties, such as adopting evidence-based practices.

**Table 1:** Specific aims, research questions, hypotheses, and data sources (N ~ 150 unless specified).

Study Aim	Research Question(s) or Hypothesis	Data Source(s)
1. Engage, recruit, and conduct needs assessments with 150 primary care clinics and their CCOs within the state of Oregon	*RQ 1a*. *How do CCOs work with small to medium sized clinics to achieve OHA’s SBIRT quality incentive metric*?	• Interviews with CCO leaders (N = 15) • Clinic contact logs • Baseline assessment: clinic intake form (including the SBIRT and MAUD implementation checklist), needs assessment, and HIT capacity assessment.
	*RQ 1b*. *How are clinics approaching SBIRT and MAUD and what is their capacity for quality metric reporting*?	
2. Implement and evaluate the impact of foundational and supplemental implementation support on SBIRT, MAUD, and QI capacity in participating primary care clinics.	*Hyp 2a*: *Clinics with a prior relationship with ORPRN or warm handoff from their CCO will be more likely to engage with and complete all study elements*. *(ADOPTION*, *MAINTENANCE)* *Hyp 3b*: *Practice facilitation to improve QI capacity and increase SBIRT and MAUD will be most effective in clinics with a QI lead*, *clinic champion and routine performance data review*. *(REACH*, *EFFECTIVENESS)*	• Supplemental implementation support plans • Clinic contact logs • Periodic reflections with practice facilitators • Interviews with clinic primary point of contact (N = 150) and practice facilitators • Baseline and exit clinic intake form • Quantitative data on clinics’ SBIRT and MAUD performance
3. Describe how practice facilitators tailor implementation support based on context, intervention characteristics, and personal expertise using mixed-methods and systems science.	*RQ 3a*. *What is the spectrum of tailored implementation support that practice facilitators provide across the participating primary care clinics*?	• Baseline assessment • Clinic contact logs • Supplemental implementation support plans • Periodic reflections with practice facilitators • Interviews with clinic primary point of contact (N = 150) and practice facilitators
	*RQ 3b*. *How do practice facilitators decide to tailor implementation support based on the innovation*, *context*, *and recipients and how does this expertise develop over time*?	

CCOs = Coordinated Care Organizations; SBIRT = Screening, brief intervention, and referral to treatment; OHA = Oregon Health Authority; MAUD = Medication-assisted treatment for alcohol use disorder; ORPRN = Oregon Rural Practice-based Research Network; HIT = Health information technology; QI = Quality improvement

### Conceptual framework

**Fig 1:**
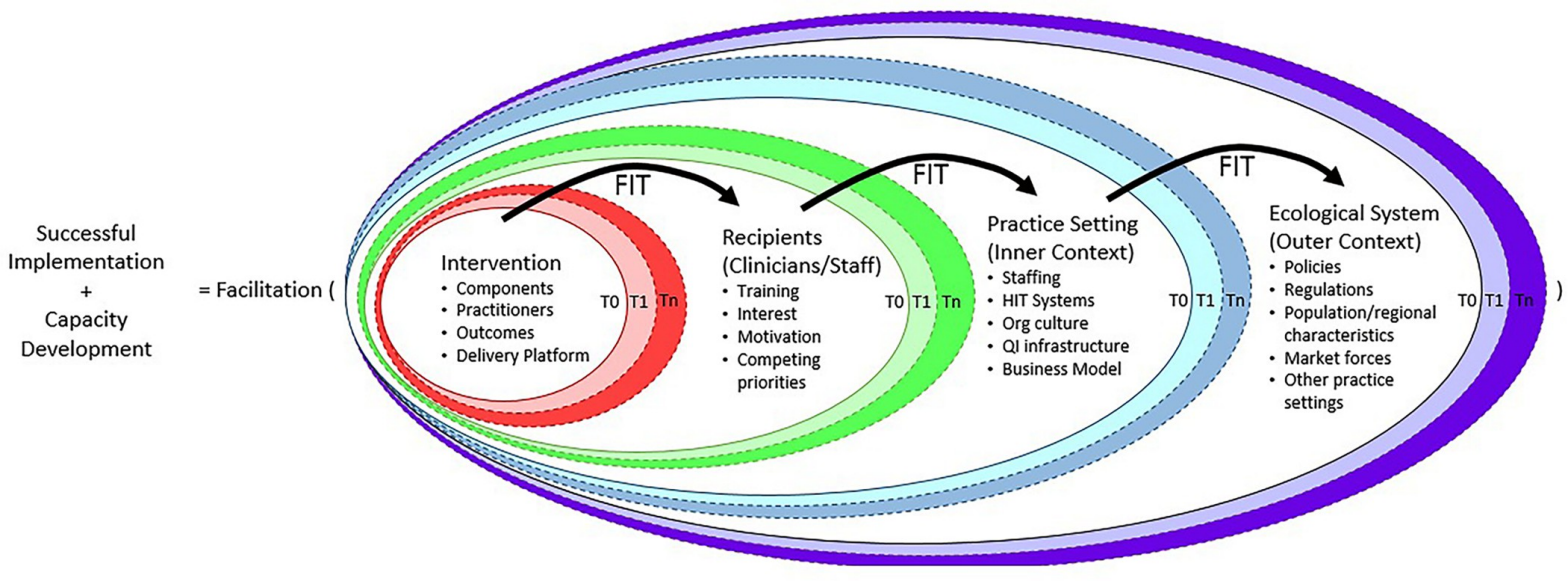
Hybrid framework combining the Integrated Promoting Action on Research Implementation in Health Services (i-PARIHS) and the Dynamic Sustainability Framework (DSF). Successful implementation and capacity development are a function of facilitation interacting with the intervention, recipients, practice setting, and the ecological system over time (represented by T0, T1,…,Tn), each of which has constituent components that may vary. HIT = Health information technology; org = Organization; QI = Quality improvement.

ANTECEDENT applies a conceptual framework that is a hybrid of the integrated Promoting Action on Research Implementation in Health Services (i-PARIHS) [33] and the Dynamic Sustainability Framework (DSF) (Fig 1) [34]. The i-PARIHS framework focuses on practice facilitation to align implementation support to the innovation, recipients, and broader contexts (e.g., local, regional) [33]. The DSF highlights the longitudinal nature of change, such that interventions are adapted and improved as they integrate into real-world settings over time [34]. Our hybrid framework allows us to understand practice facilitation as a meta-strategy for implementation support and explore how practice facilitators work with clinics over time to support SBIRT and MAUD delivery in routine primary care.

### Clinic eligibility and recruitment

Any clinic in Oregon providing primary care to adult patients is eligible for the study. Prior work by our team identified approximately 750 primary care clinics in Oregon that meet eligibility criteria; we aim to engage approximately 20% of potentially eligible sites [35]. ANTECEDENT uses a rolling recruitment process to capitalize on changing capacity and interest in clinics, health systems, and CCOs over time. Clinic recruitment begins with engagement and outreach to regional CCOs and clinics with established ORPRN relationships. Prior research in Oregon suggests that larger and system-affiliated clinics are prioritized for support by the CCOs [36]. Thus, recruitment activities focus on reaching small- to medium-sized clinics (defined as clinics with fewer than 10 primary care providers) and independent clinics.

### Evidence-based intervention

Evidence-based interventions are the programs, policies, or procedures integrated into routine practice [37]. These are the necessary steps to improve care in a specific setting [38]. As summarized in Table 2, the U.S. Preventive Services Task Force (USPSTF) and Cochrane Collaboration recommend that clinicians screen adults for UAU and provide brief interventions to individuals engaged in risky drinking [5, 30, 31, 39]. While referral to treatment is not currently the national recommendation [40, 41], it is identified as a qualifying intervention within Oregon’s CCO quality metric for SBIRT [27]. To align with this state-level context, we tailored our intervention approach to support SBIRT for UAU and recreational and illicit drug use.

**Table 2:** Evidence-based interventions recommended for addressing unhealthy alcohol use in primary care.

Topic	Identified In	Key Finding
Screening	USPSTF	“The USPSTF recommends screening for unhealthy alcohol use in primary care settings in adults 18 years or older, including pregnant women…” Furthermore, “1-item to 3-item screening instruments have the best accuracy for assessing unhealthy alcohol use in adults 18 years or older.” These include the *Alcohol Use Disorders Identification Test-Consumption* and the National Institute on Alcohol Abuse and Alcoholism-recommended *Single Alcohol Screening Question* [5].
Brief Intervention	Cochrane Collaboration	Brief intervention (BI) for alcohol misuse consistently produced reductions in alcohol consumption. At follow-up one year later, people who had received BIs drank 6–25g less alcohol per week. BIs include feedback on alcohol use and harms, identification of high-risk situations for drinking and coping strategies, increased motivation and the development of a personal plan to reduce drinking. BIs involve one to four sessions and take place within the timeframe of a standard office visit. Longer counselling showed little additional benefit [39].
	USPSTF	The USPSTF recommends… “providing persons engaged in risky or hazardous drinking with brief behavioral counseling interventions to reduce unhealthy alcohol use.” There are no specific intervention characteristics or components that are clearly associated with improved outcomes [5].
Medications for Alcohol Use Disorder	Cochrane Collaboration	The opioid antagonist naltrexone supports cutting down on drinking through reducing alcohol “liking” and “craving”. It reduces risk of returning to heavy drinking (5/4 standard drinks daily) to 83% of placebo, and decreases drinking days by around 4%. NNT = 9 for not returning to heavy drinking [31].
		Compared to placebo, Acamprosate and psychosocial treatment significantly reduce the risk of any drinking (NNT = 9) as well as increasing the cumulative duration of abstinence. Side effects did not cause subjects to stop treatment any more than placebo [30].

USPSTF = United States Preventive Services Task Force; NNT = Number needed to treat

### Implementation support

Implementation strategies are the methods or means by which target interventions are put into routine practice [38, 42]. Implementation strategies can be delivered as discrete strategies or bundles (e.g., a package of discrete concepts designed to address specific barriers and support implementation success) [38]. Facilitation is a multi-component implementation strategy [43]. As summarized in Table 3, ANTECEDENT implementation support is coordinated by a practice facilitator and uses a two-phase approach aligned with Proctor’s recommendations for reporting implementation strategies [42]. S1 Table and S2 Appendix provide additional detail about the timing and content of the implementation strategies, respectively.

**Table 3:** Implementation support strategies.

*Implementation Strategy*	*Definition*	*Dimension*				
		*The Actor(s)*	*The Action(s)*	*Action Targets*	*Temporality*	*Dose*
Foundational Support						
Baseline Assessment	Assessment of capacity, interest, and needs	Practice facilitators	Evaluate context, HIT capacity, and inform supplemental support	QI leads and care team	At baseline (recruitment)	One time: four to six hours
Implementation Toolkit	Implementation guide for SBIRT	SBIRT Oregon website	Provide asynchronous access to toolkit, resources, and training materials	Primary: QI leads Secondary: Practice facilitators	Ongoing	Number of visits and reported utilization
e-Screening SBIRT tool	Evidence-based tool for screening	Care team members	Provide a clinical decision support screener	Care team members	Ongoing	Number of documented screens
Exit Assessment	Assessment of capacity and impact	Practice facilitators	Evaluate context and produce data reports	QI leads and care team	After intervention	One time: three to five hours
Supplemental Support						
Practice facilitation	Process of interactive problem solving and support	Practice facilitators	Engage leadership; assess workflows; assist with changes; monitor and encourage progress	QI leads	Six months: two hours of (in person and/or virtual) practice facilitation per month	Visit length and intensity are tailored: up to 10 hours direct practice facilitator support
HIT support	HIT experts help with data extraction, entry, and review	HIT experts and practice facilitators	Design reports; help practice facilitators teach clinics to run reports and document data	QI leads and/or clinic QI teams; practice facilitators	At start of study, ongoing if needed	Up to five hours
Audit and Feedback	Performance data provided for QI	Practice facilitators or QI leads	Audit process measures regarding patient service engagement	QI leads and care team members	Monthly, when possible	Not applicable
Peer-to-Peer Learning via Webinars	Education regarding SBIRT elements	Co-investigators	Provide virtual training regarding patient-centered outcomes research evidence and workflows	QI leads and care team members	Live webinars during year one; recordings thereafter	Up to three hours of webinars
Peer-to-Peer Learning via Oregon ECHO^®^ Network	Education and reflection regarding MAUD	Co-investigators	Provide telemedicine training for MAUD.	Primary care clinicians	Six sessions over 5 months during year two	Up to six, 90-minute sessions
Expert Consultation	Experts in SBIRT, MAUD changes	Co-investigators	Discuss patient-centered outcomes research evidence; review SBIRT metric and workflows	QI leads and/or care team members	As needed to support facilitators	Up to two hours direct practice support

HIT = Health information technology; QI = Quality improvement; SBIRT = Screening, brief intervention, and referral to treatment; EHR = Electronic health record; MAUD = Medication-assisted treatment for alcohol use disorder; ECHO^®^ = Extension for Community Healthcare Outcomes

#### Foundational support

All participating clinics receive foundational support, which allows collecting a minimum data set needed for the evaluation. Foundational support includes a baseline assessment (first three project months), SBIRT Oregon online toolkit access, and an exit assessment (final three project months). The SBIRT Oregon online toolkit provides multiple resources, including demonstration videos, role-play exercises, clinic workflows guides, pocket-sized readiness rulers, and patient testimonial videos [12]. Our team anticipates that with access to SBIRT Oregon resources, certain highly motivated clinics will be able to improve their SBIRT workflows and reporting.

#### Supplemental support

Prior work highlights dedicated staff’s value in supporting and implementing guidelines and toolkit resources into clinical workflows and practices [15, 44, 45]. Thus, we also seek to provide supplemental implementation support, coordinated by a trained practice facilitator, to interested clinics. Supplemental support may include practice facilitation, HIT support, peer-to-peer learning collaboratives, and expert consultation via academic detailing. Academic detailing is an interactive educational outreach to providers and staff to disseminate evidence-based information about prescribing practices and therapeutic decision-making to improve patient care [46]. HIT support includes generating data to inform changes to workflows and reporting for Oregon’s CCO SBIRT metric [27]. If the Oregon CCO metric cannot be generated or is not desired by the clinic, the practice facilitator will explore alternative measures, including the National Quality Forum 2152/ Centers for Medicare & Medicaid Services 431 or help develop a custom EHR query [47]. See Table 3 and S2 Appendix for additional detail on the supplemental support strategies.

### Data sources and collection

As detailed in Table 4, we will use qualitative data from key informant interviews, periodic reflections, clinic surveys, clinic contact logs, and quantitative measures from clinic assessments to address our research aims. In addition to addressing the aims proposed by our ANTECEDENT team, we will also gather the data required to contribute to the cross-site evaluation for all AHRQ EvidenceNOW UAU grantees. Study data is collected and managed using REDCap (Research Electronic Data Capture) tools, a secure, web-based software platform designed to support data capture for research studies [48, 49].

**Table 4:** Planned data sources, collection strategies, and frequency.

*Data*	*Purpose*	*Source of Data*	*Timing / Frequency* ^a^ ^b^
CCO key informant interviews	To understand how CCOs work with clinics to improve SBIRT and MAUD workflows and documentation, inform baseline understanding or regional context, and to support clinic recruitment.	Interviews with CCO leaders	Within first 12 months of project funding
Clinic contact logs	To monitor clinic engagement, implementation support, changes in context or recipients, adaptations to the intervention, and changes to care delivery.	Practice facilitators complete in REDCap	Following all clinic interactions
Clinic intake Form—Includes SBIRT and MAUD checklist	To describe practice size, staffing, patient population, telehealth utilization, EHR system at time of enrollment. The clinic intake form includes an SBIRT and MAUD checklist used across all grantee programs. This checklist will help determine clinic capacity to capture and report data on intervention outcomes, delivery protocols and inform tailored EHR support.	Survey (online or paper)	Baseline and exit assessment
Needs assessment	To understand clinic interest in and experience with SBIRT, MAUD, and QI, and to inform tailored implementation support.	Pre-intervention meeting with clinics	Baseline assessment
Workflow observation	To understand how clinics deliver SBIRT and MAUD at baseline; to identify promising leverage points.	Observation by practice facilitator	Baseline assessment
HIT capacity assessment	To determine clinic capacity to report SBIRT and MAUD measures; to inform tailored implementation support.	Clinic completes via survey and conversation with practice facilitator	Baseline assessment
Supplemental implementation support plan	To lay out anticipated implementation support plan for each clinic.	Developed by practice facilitator	Within four weeks of clinic baseline assessment
Practice facilitator key informant interviews	To explore how individual facilitators tailor implementation support and the development of personal expertise.	Interviews with practice facilitators	Every six months
Periodic group reflections with practice facilitators	To explore how facilitators tailor implementation support, identify patterns in implementation across clinics, and track adaptations.	Facilitated debriefs with practice facilitators	Monthly
SBIRT and MAUD performance data collection	To identify the proportion of eligible patients receiving SBIRT and MAUD (outcome dependent variable) and ability to report the Oregon CCO metric to their regional Medicaid organization.	EHR or manual tracking	Baseline and exit assessment
Clinic key informant interviews	To understand how clinics experience implementation support and practice facilitator development over time; to understand tailoring of implementation support and facilitator skill development.	Interviews with clinic primary point of contact	Exit assessment

^a^ Baseline assessment activities occur within the first three months of project initiation

^b^ Exit assessment activities occur within the last three months of project support (months 13–15)

CCOs = Coordinated Care Organizations; SBIRT = Screening, brief intervention, and referral to treatment; MAUD = Medication-assisted treatment for alcohol use disorder; HIT = Health information technology; REDCap: Research Electronic Data Capture; EHR = Electronic health record

#### Key informant interviews

We will conduct semi-structured qualitative interviews with three types of key informants: CCO staff, practice facilitators, and clinic primary points of contact. Interviews with CCO staff (N = 15), conducted in the first 12 months of the study, will address how CCOs work with clinics to improve their workflows and documentation for delivery of SBIRT and MAUD, informing a baseline understanding of regional context and supporting clinic recruitment. Every six months, individual semi-structured interviews will be conducted with practice facilitators to gather information about how they tailor implementation support to clinics and develop their expertise. Exit interviews with the clinic primary point of contact will be conducted following implementation. They will provide data about the clinic’s experience with implementation support and the status of their SBIRT performance at the conclusion of the study. All interviews will last 45–60 minutes and will be conducted by qualitative analysts in person or via videoconference. Transcripts will be professionally transcribed and validated for accuracy.

#### Clinic contact logs

Following all clinic interactions, practice facilitators will record field notes in REDCap using structured forms containing open- and closed-ended prompts. Practice facilitators will use two types of structured note-reporting guides to record their interactions and clinic support. Brief communication logs will track short interactions with clinics (e.g., email outreach, scheduling), and in-depth intervention tracker forms will describe engagement, content, and the quality of scheduled facilitation meetings. The logs will produce evaluation data specified in the request for applications, such as the number and type of interactions and implementation strategies employed. The intervention tracker documents practice facilitator tailoring concerning clinic context and perceptions of clinic capacity for change. A qualitative analyst will regularly review the clinic contact logs for completeness.

#### Clinic baseline assessment

The baseline assessment includes five data points: The clinic intake form, needs assessment, workflow observations, HIT capacity assessment, and SBIRT and MAUD performance data collection (Table 4). The clinic intake form is a survey administered to collect details on clinic size, ownership, and electronic health record use. The clinic intake form includes an SBIRT and MAUD implementation checklist. This checklist will help determine a clinic’s capacity to capture and report data on intervention outcomes and inform tailored EHR support. The needs assessment addresses clinic practice culture, external context, and QI capacity. The observation of practice workflows will involve shadowing primary care clinicians and clinic staff to understand clinic culture and workflows related to SBIRT and MAUD. The HIT capacity assessment is used to gauge a clinic’s screening and tracking processes and ability to report the CCO SBIRT metric. In tandem, we will collect SBIRT and MAUD performance data using aggregate quantitative data from clinic EHRs or via manual chart audits. These final two data sources will inform the level of HIT support clinics receive to produce study outcomes data and CCO SBIRT quality metric reporting.

#### Supplemental implementation support plan

Practice facilitators use information from the baseline assessment to develop and refine a tailored supplemental support plan for each clinic in partnership with the clinic’s primary point of contact [50]. This plan draws on findings from the needs assessment. It specifies the anticipated supplemental implementation strategies that the clinic receives (e.g., practice facilitation, HIT support, and expert consultation for SBIRT or MAUD workflows) and whether the activities will occur remotely or in person. The supplemental support plan is delivered over a 9-month implementation period. Supplemental implementation support plans may continue to evolve based on the clinics’ needs and capacity during the active facilitation period; the initial plan and adaptations will be documented in the REDCap database by the practice facilitator.

#### Periodic reflections with practice facilitators

Monthly, a qualitative analyst will facilitate 60-minute periodic reflections with the practice facilitator group via videoconference [51]. The qualitative analyst will solicit discussion topics from study leadership and practice facilitators before each session and develop a short interview guide. The group format will help identify patterns across clinics and facilitators related to clinic context and tailoring of implementation support. Sessions will be recorded, professionally transcribed, and validated for accuracy.

#### Exit assessment

All clinics will participate in an exit consultation, coordinated by the practice facilitator, in the final quarter of the intervention (months 13–15). The closing activities allow solidifying program maintenance and updating the clinic intake form (including the SBIRT and MAUD implementation checklist). During the exit assessment, we will also collect the second set of SBIRT and MAUD performance data to compare SBIRT and MAUD delivery rates to eligible patients at baseline and post-intervention per the specifications of the request for applications and external evaluation. We anticipate that some clinics will develop the capacity for baseline reporting via participation in the intervention. After completing these activities, the practice facilitator will connect the clinic point of contact to the qualitative team to complete the study exit interview. Additional information on the clinic intake form (baseline assessment) and exit interview (key informant interviews) are provided above and in Table 4.

### Data analysis

#### Quantitative analytic plan

Quantitative data will be analyzed using Stata. We propose two primary performance outcomes. The first is the difference between baseline and exit assessment in the percentage of eligible patients who receive screening for UAU (and drug risk when using Oregon’s SBIRT protocol), brief intervention, and MAUD. Because baseline levels may be strongly correlated with change, we also plan to conduct a sensitivity analysis to scale the change as a proportion of the initial gap between baseline and target. The second outcome is whether the clinic can report the Oregon CCO SBIRT metric to its regional CCO by the end of the study. These outcomes will serve as the dependent variable of a linear (percentage difference) or logistic (ability to report CCO measure) regression model.

To evaluate the impact of tailored implementation support on SBIRT, MAUD, and QI capacity (Aim 2), we will combine data on clinics’ previous collaborations with ORPRN, how clinics were referred to ANTECEDENT and the number and types of contacts in the recruitment and implementation phases. We will use logistic regression to determine which factors increase the probability of successful recruitment and project completion. In addition to the predictors above, we will account for the possibility that the level of implementation success depends on the specific practice facilitator or CCO. We anticipate that higher-intensity contact will be negatively associated with clinic enrollment but positively associated with study completion (defined as participation in exit assessment). We further hypothesize that implementation support will be most effective when clinics have a QI lead and clinic champion.

#### Qualitative analytic plan

All qualitative data will be maintained in ATLAS.ti and analyzed to identify patterns or themes according to Miller and Crabtree’s editing method [52, 53]. Our theoretical framework, a hybrid of i-PARIHS [33] and DSF [34] (Fig 1), will inform coding and analysis. Transcripts from the interviews with CCO staff will be coded and analyzed concurrently with data collection to identify how CCOs support clinics in achieving the SBIRT quality incentive metric (Aim 1). The remaining qualitative data (interviews with clinic contacts and PERCs, clinic contact logs, clinic surveys, baseline assessment, and periodic reflection transcripts) will be coded and analyzed on a rolling basis as clinics conclude participation in the intervention. Multiple coders will analyze, discuss data, and synthesize findings of clinic context, QI capacity, engagement with the intervention, level of implementation support provided, clinic disruptions, and ability to capture and report quantitative performance data on SBIRT and MAUD. In addition to clinic-level analysis, qualitative data about practice facilitator expertise and perspectives of practice transformation will be analyzed concerning study Aim 3. Preliminary themes at both levels will be identified and refined in consultation with the study team and ANTECEDENT Advisory Board as a form of member checking [52, 53].

#### Systems science analytic plan

To examine how practice facilitators tailor implementation support based on clinic context, the SBIRT innovation, and expertise over time, we will systematically generate causal-loop diagrams from qualitative data to describe changes in practice facilitators’ perspectives of practice transformation (Aim 3). Causal-loop diagramming is a method from systems science that identifies feedback loops driving nonlinear behavior in complex systems [54, 55]. Diagrams corresponding to each facilitator’s perspective will allow for comparison between individuals over time. Once data collection is complete, we will use a group modeling approach [56] to synthesize the team’s mental model of effective practice transformation to support SBIRT and MAUD in primary care.

#### Mixed-methods convergence

Finally, we will merge our quantitative, qualitative, and systems science data using joint displays to reconcile differences and enable the identification of patterns and themes across clinics [57]. To further examine implementation support provided to clinics (Aim 2), we will review descriptive statistics from the clinic contact logs, clinic intake form, and HIT capacity assessment in tandem with our qualitative data. Our goal is to classify clinics along three primary dimensions: (1) baseline capacity, (2) level of implementation support, and (3) observed improvement, and to examine details of the relationships among them. To define baseline capacity, we will categorize clinics as “high”, “medium”, or “low” based on QI infrastructure and workflows for SBIRT and MAUD during the baseline assessment. Qualitative and quantitative analysts will conduct a card sorting activity to classify the participating clinics based on the level of implementation needed to implement SBIRT and MAUD successfully. Analysts will use these resulting categories to create causal predictive matrices to describe clinic features and understand the factors associated with improvement [58]. We will use correspondence analysis to investigate relationships between categorical variables (for example, whether the distribution of implementation support activities varies with clinic baseline capacity) and latent class analysis to identify groups of clinics with similar characteristics. These methods represent high-dimensional data in a low-dimensional space so that it is easier to identify patterns and critical features [59]. We hypothesize that depending on the clinic context, varying levels of implementation support may be needed to support “successful implementation”. We will strive to identify case examples to inform studies using predictive models for tailored implementation support.

#### Sample size considerations

Recruiting 150 clinics will ensure we retain a minimum of 120 clinics in the final analysis after an anticipated attrition rate of 20% based on prior studies. While we lack preliminary performance estimates for SBIRT for UAU to inform power calculations, for a similar measure—depression screening and follow-up—the OHA reported that CCO performance in the first year of tracking (2014) ranged from 3.3% to 68.1% [60]. Thus, we anticipate similar, significant variability in clinic-level performance outcomes. Our sample of 120–150 clinics ensures that even targeted sub-group analyses performed at the clinic level will have desirable “large” sample properties [59].

## Discussion

ANTECEDENT is a pragmatic implementation study designed to support primary care clinics in implementing and refining workflows to support SBIRT and MAUD as part of routine care. Our study bridges improvement science, implementation science, and participatory methods, allowing for local change and the production of generalizable knowledge [18, 61]. The mixed-methods analysis will advance our understanding supporting program implementation across diverse primary care contexts and how practice facilitators tailor implementation support to context over time [14, 43, 62]. ANTECEDENT intervention activities commenced in February 2020, just before multiple changes in clinical practice and research protocols in response to the COVID-19 pandemic [63–65]. Adaptations in response to the pandemic and the impact on study activities will be addressed in subsequent publications, allowing us to expand our understanding of how to adapt primary care research and implementation support to clinics during crises.

Many prior studies focus on implementing SBIRT in integrated settings with high-functioning EHRs [4, 66–69]. ANTECEDENT fills a gap in research and regional improvement by focusing recruitment and implementation on small- to medium-sized clinics [27, 36]; we anticipate reaching clinics with multiple EHR systems with varied functional capacity. Therefore, ANTECEDENT has the potential to both reach patients most in need of UAU interventions and to advance our understanding of the levels needed to support clinic change effectively.

## Supporting information

S1 Appendix Screening, brief intervention, and referral to treatment as a quality incentive metric for Coordinated Care Organizations in Oregon. (PDF)

S2 Appendix Detailed implementation strategies. (PDF)

S1 Table ANTECEDENT study timeline. ANTECEDENT (p**A**rt**N**erships **T**o **E**nhance al**C**ohol scr**E**ening, treatment, an**D** int**E**rve**NT**ion); IRB = Institution Review Board; CCOs = Coordinated Care Organizations; SBIRT = Screening, brief intervention, and referral to treatment; MAUD = Medication-assisted treatment for alcohol use disorder.
(PDF)

## Abbreviations

UAU: Unhealthy alcohol use
SBIRT: Screening, brief Intervention, referral to treatment
MAUD: Medication-assisted treatment for alcohol use disorder
ORPRN: Oregon Rural Practice-based Research Network
CCO: Coordinated Care Organization
QI: Quality improvement
RE-AIM: Reach, effectiveness, adoption, implementation, maintenance
i-PARIHS: Integrated Promoting Action on Research Implementation in Health Services
DSF: Dynamic Sustainability Framework
OHA: Oregon Health Authority
AHRQ: Agency for Healthcare Research and Quality
PBRN: Practice-based research network
IRB: Institutional Review Board
EHR: Electronic health record
HIT: Health information technology
REDCap: Research Electronic Data Capture
Org: Organization
USPSTF: United States Preventive Services Task Force
NNT: Number needed to treat
