# Supplementary material for: Improving screening, treatment, and intervention for unhealthy alcohol use in primary care through clinic, practice-based research network, and health plan partnerships: Protocol of the ANTECEDENT study
Source: PLoS One. 2022 Jun 28;17(6):e0269635. doi: 10.1371/journal.pone.0269635 (PMC9239445; doi:10.1371/journal.pone.0269635)
Supplement: S1 Appendix — (PDF) [file pone.0269635.s001.pdf]

S1 Appendix. Screening, brief intervention, and referral to treatment as a quality incentive metric for Coordinated Care Organizations in Oregon.

Screening, brief intervention, and referral to treatment (SBIRT) have been included as an annual quality metric (https://www.oregon.gov/oha/HPA/ANALYTICS/Pages/CCO-Metrics.aspx) for Oregon Coordinated Care Organizations (CCOs) for multiple years, and in multiple assessment strategies over time. In 2013, Oregon Health Authority (OHA) included SBIRT in the pay-for-performance incentive metrics used to evaluate the quality of care delivered to Medicaid patients by CCOs. By 2016, the CCO SBIRT incentive metric met with partial success, resulting in increased screenings for unhealthy alcohol and drug use, as evidenced by billing codes. However, it did not increase alcohol or drug use disorder diagnosis codes or the initiation of treatment, suggesting that the state policy did not fully accomplish its purpose [1]. The SBIRT quality incentive metric was removed in 2017, revised throughout 2018, and was reintroduced as an electronic health record level reported incentive metric for Oregon's CCOs in January 2019. Most recently, OHA added reporting benchmarks in 2022 for the SBIRT quality incentive metrics [2].

References

1. Rieckmann T, Renfro S, McCarty D, Baker R, McConnell KJ. Quality metrics and systems transformation: Are we advancing alcohol and drug screening in primary care? Health Serv Res. 2018;53(3):1702-26.
2. Oregon Health Authority. CCO metrics 2022 [cited 2022 March 24]. Available from: https://www.oregon.gov/oha/HPA/ANALYTICS/Pages/CCO-Metrics.aspx.
