# Supplementary material for: Improving screening, treatment, and intervention for unhealthy alcohol use in primary care through clinic, practice-based research network, and health plan partnerships: Protocol of the ANTECEDENT study
Source: PLoS One. 2022 Jun 28;17(6):e0269635. doi: 10.1371/journal.pone.0269635 (PMC9239445; doi:10.1371/journal.pone.0269635)
Supplement: S2 Appendix — (PDF) [file pone.0269635.s002.pdf]

S2 Appendix. Detailed implementation strategies

Foundational support

Baseline assessment

We will conduct the baseline assessment within four to eight weeks of clinic recruitment to ANTECEDENT. The baseline assessment takes four to six hours to complete. It provides facilitators with a basic understanding of practice culture, capacity, and the external context in which the clinic operates. Baseline assessment includes the following:

- ***Clinic information form*** gathers details on clinic size, ownership, EHR system features, and QI structure. This form includes the SBIRT and MAUD implementation checklist. This assessment takes 15-30 minutes to complete.
- ***Needs assessment*** is a 30 to 60-minute interview designed to understand elements in our hybrid conceptual framework (organizational culture, external context), the clinic's motivation for participating the in the project, current practices around unhealthy alcohol use, the clinic's prior experience with facilitation and QI projects, and to orient them to the study formally.
- ***Observation of practice workflows*** will take approximately three hours. It will include shadowing primary care clinicians, front desk staff, and back-office staff to understand clinic culture and workflows related to SBIRT and MAUD. The facilitator takes detailed jottings and will prepare rich field notes within 24 hours.
- ***HIT capacity assessment*** to determine current clinic screening and tracking processes and clinic quality metric capacity reporting for the CCO SBIRT metric, the ANTECEDENT evaluation, and the level of HIT support needed.

SBIRT implementation toolkit

Several resources (e.g., screening tools, patient education materials, motivational interviewing aides, and video demonstrations) to support SBIRT for drug and alcohol use exist on the SBIRT Oregon website. These materials are foundational and can be used immediately should a site have adequate leadership and QI capacity to support change. Additionally, ANTECEDENT will update these materials with new patient-centered outcomes research evidence and details on MAUD. Exit assessment

This assessment will be used to update data collected in the baseline assessment (e.g., quantitative performance data on the delivery of screening, brief interventions, and MAUD, updates to the clinic information form, and the key informant interviews with clinic primary point of contact).

Supplemental support

The practice facilitator will use information from the baseline assessment to create a gap analysis and recommend strategies for tailored supplemental implementation support. The ANTECEDENT team will review this supplemental support plan and finalize it with the clinic's primary point of contact at each participating clinic. Based on our prior experience, we anticipate that about half of the sites will want intensive support, and the other half will want occasional consultations and access to project resources. We anticipate that the supplemental support will last approximately six months for each site. This will include a minimum of two phone check-ins and a maximum of four in-person visits (in addition to the two in-person visits by the practice facilitator that will occur for the baseline and exit assessments).

Practice facilitation

Facilitation is used as a meta-strategy to support implementation and build capacity, as detailed in our hybrid conceptual framework. While toolkits can help with practice change, practice facilitation is often critical in translating these resources into practice [1]. ORPRN practice facilitators are trained in research, data collection methods, and QI activities, such as conducting workflow mapping, setting Plan-Do-Study-Act cycles and guiding the selection of data-driven QI goals that support key drivers [2]. Facilitators will work with QI leads from each site to apply change strategies for these key drivers, set improvement targets and monitor progress. Facilitators may address multiple key drivers simultaneously.

HIT support

Based on findings from the baseline assessment, clinics may be eligible for up to 10 hours of HIT support to help clinics develop their data entry and reporting capacity for the SBIRT metrics. Our team has been able to help clinics solve most HIT issues with remote technical assistance. ORPRN practice facilitators have expertise with several common EHRs, and will receive support from a Senior Research Associate with clinical informatics expertise.

Audit and feedback

We will work with clinics to produce summaries of clinical performance related to SBIRT and MAUD at the provider/team level. We will target monthly data reporting and review to inform improvement goals as frequent feedback on performance is often associated with change and sustained improvement [3].

Peer-to-peer learning

Allowing stakeholders to interact and learn from each other is an effective strategy for implementation support. In ANTECEDENT, we will utilize webinars and Project ECHO® (Extension for Community Healthcare Outcomes) to help clinics and clinicians implement SBIRT and MAUD. We will collaborate closely with other TA providers and the OHA Transformation Center to advertise and deliver this support.

- ***Webinars*** related to the SBIRT CCO quality metric, and implementing SBIRT and MAUD workflows will be led by two ANTECEDENT co-investigators. These webinars will be delivered in project year one and posted on the SBIRT Oregon website for asynchronous viewing.
- ***Oregon ECHO® Network*** will hold a six-session ECHO® over five months in year 2 related to alcohol abuse, SBIRT, MAUD and the CCO SBIRT metric. Several addiction-related six to 12-week ECHO® programs exist and have successfully engaged more than 200 participants in Oregon. Each ECHO® session includes a 15-minute expert presentation on a topic followed by an interactive case discussion.

Expert consultation

Two co-investigators will be available to travel to clinics and participate in virtual calls to troubleshoot areas related to SBIRT and MAUD implementation. These meetings will be coordinated through the ORPRN practice facilitator based on identified clinic needs.

References

1. Davis MM, Howk S, Spurlock M, McGinnis PB, Cohen DJ, Fagnan LJ. A qualitative study of clinic and community member perspectives on intervention toolkits: "Unless the toolkit is used it won't help solve the problem". BMC Health Serv Res. 2017;17(1):497.
2. Michaels L, Anastas T, Waddell EN, Fagnan L, Dorr DA. A randomized trial of high-value change using practice facilitation. J Am Board Fam Med. 2017;30(5):572-82.
3. Ivers N, Jamtvedt G, Flottorp S, Young JM, Odgaard-Jensen J, French SD, et al. Audit and feedback: effects on professional practice and healthcare outcomes. Cochrane Database Syst Rev. 2012(6):CD000259.
