# Supplementary material for: Improving screening, treatment, and intervention for unhealthy alcohol use in primary care through clinic, practice-based research network, and health plan partnerships: Protocol of the ANTECEDENT study
Source: PLoS One. 2022 Jun 28;17(6):e0269635. doi: 10.1371/journal.pone.0269635 (PMC9239445; doi:10.1371/journal.pone.0269635)
Supplement: S1 Table — ANTECEDENT (pArtNerships To Enhance alCohol scrEening, treatment, anD intErveNTion); IRB = Institution Review Board; CCOs = Coordinated Care Organizations; SBIRT = Screening, brief intervention, and referral to treatment; MAUD = Medication-assisted treatment for alcohol use disorder. (PDF) [file pone.0269635.s003.pdf]

S1 Table. ANTECEDENT study timeline

	Pre-Award	Year 1				Year 2				Year 3			
		Q1	Q2	Q3	Q4	Q1	Q2	Q3	Q4	Q1	Q2	Q3	Q4
IRB and practice facilitation training	X	X											
Patient-centered outcomes research evidence updates	X	X	X	X	X	X	X	X	X	X	X	X	X
CCO interviews	X	X	X										
Clinic recruitment (rolling start)			X	X	X	X	X	X	X	X			
Baseline assessment (three months)			X	X	X	X	X	X	X	X			
Tailored implementation support (nine months)				X	X	X	X	X	X	X	X	X	
Exit assessment (three months)						X	X	X	X	X	X	X	X
Quantitative performance data on the delivery of SBIRT and MAUD (with exit consultation)						X	X	X	X	X	X	X	X
Monthly practice facilitator debriefs			X	X	X	X	X	X	X	X	X	X	
Interviews with clinic primary point of contact						X	X	X	X	X	X		
Quantitative data analysis					X	X	X	X	X	X	X		
Qualitative, mixed methods and systems science data analysis			X	X	X	X	X	X	X	X	X	X	
Dissemination		X	X	X	X	X	X	X	X	X	X	X	X

ANTECEDENT=p**A**rt**N**erships **T**o **E**nhance al**C**ohol scr**E**ening, treatment, an**D** int**E**rve**NT**ion; IRB=Institution Review Board; CCOs=Coordinated Care Organizations; SBIRT=Screening, brief intervention, and referral to treatment; MAUD=Medication assisted treatment for alcohol use disorders
